# Prosthesis-socket volume imbalance and dermofat graft rehabilitation in patients with an anophthalmic socket

**DOI:** 10.5935/0004-2749.20200003

**Published:** 2020

**Authors:** Selam Yekta Sendul, Burcu Dirim, Cemile Ucgul Atılgan, Mehmet Demir, Semra Tiryaki Demir, Ali Olgun, Saniye Uke Uzun, Murat Karapapak, Dilek Guven

**Affiliations:** 1 Department of Ophthalmology, Sisli Hamidiye Etfal Training and Research Hospital, Istanbul, Turkey; 2 Department of Ophthalmology, Ulucanlar Goz Training and Research Hospital, Ankara, Turkey

**Keywords:** Anophthalmos, Orbital implants, Dermis, Prosthesis and implants, Ophthalmologic surgical procedures, Anoftalmia, Implantes orbitários, Derme, Próteses e implantes, Procedimentos cirúrgicos oftalmológicos

## Abstract

**Purposes:**

To identify problems caused by prosthesis-socket volume imbalances in anophthalmic sockets; and to evaluate rehabilitation with dermofat graft as a solution.

**Methods:**

We retrospectively reviewed medical records of patients operated in our clinic (between May 2011 and June 2016) with dermofat grafts to treat anophthalmic socket-related problems. During the preoperative examinations, ophthalmologists recorded the presence of eyelid problems due to the socket volume deficit, upper and lower fornix deficiency, deepening in the upper eyelid sulcus, epiphora and secretion, lower eyelid laxity, ptosis, entropion, and ectropion. Following the surgical repair, new prosthesis suitable for the resulting socket area were implemented for all the patients. The mean follow-up period was 27.42±16 months (ranging from 10-62 months). On the last control examinations, ophthalmologists recorded solved and unsolved socket problems that were present preoperatively.

**Results:**

We included 16 men and 5 women in this study. The mean age was 38.3 ± 18.4 years (range, 5-75 years). The mean duration of preoperative prosthesis use was 9.4 ± 6.8 years (range, 1-30 years). Preoperatively, 7 patients had only orbital volume deficits, and 14 had socket volume displacements in addition to the volume deficits. After the dermofat graft implantations, the remaining deficits were corrected during another surgical session: 6 patients underwent ptosis corrections, 5 lateral canthal suspensions, 5 lower fornix with mucosal graft formations, and 2 upper fornix formations with mucosal grafts. All patients were able to use prosthesis postoperatively.

**Conclusion:**

The use of dermofat grafts to correct anophthalmic socket problems caused by orbital volume deficits or volume displacements is an effective, reliable, and reproducible surgical method.

## Abstract (pt)

**Objetivos:**

Identificar os problemas causados pelo desequilíbrio do volume da cavidade da prótese em cavidades anoftálmicas, e avaliar a reabilitação com enxerto de dermofato como solução.

**Métodos:**

Revisamos retrospectivamente os prontuários de pacientes operados em nossa clínica (entre maio de 2011 e junho de 2016) com enxertos de dermofato para tratar problemas relacionados a cavidades anoftálmicas. Durante os exames pré-operatórios, os oftalmologistas registraram a presença de problemas palpebrais devido ao déficit de volume, deficiência de fórnice superior e inferior, aprofundamento no sulco palpebral superior, a epífora e secreção, flacidez palpebral inferior, ptose, entrópio e ectrópio. Após a cirurgia, novas próteses adequadas para a área de encaixe foram implementadas em todos os pacientes. O tempo médio de acompanhamento foi de 27,42 ± 16 meses (variando de 10 a 62 meses). Nos últimos exames de controle, os oftalmologistas registraram problemas corrigidos e não corrigidos da cavidade que estavam presentes no pré-operatório.

**Resultados:**

Foram incluídos 16 homens e 5 mulheres neste estudo. A média de idade foi de 38,3 ± 18,4 anos (variação de 5-75 anos). A duração média do uso de prótese pré-operatória foi de 9,4 ± 6,8 anos (variação de 1 a 30 anos). No pré-operatório, 7 pacientes apresentavam apenas déficit orbitais e 14 tinham desvios de volume, além dos déficits de volume. Após os implantes de enxerto de dermoadipação, os déficits remanescentes foram corrigidos durante outra sessão cirúrgica: 6 pacientes foram submetidos a correção de ptose, 5 suspensões de cantal lateral, 5 fórnix inferior com enxerto de mucosa e 2 formações de fórnice superior com enxerto de mucosa. Todos os pacientes foram capazes de usar prótese no pós-operatório.

**Conclusão:**

A utilização de enxertos de dermofato para corrigir problemas de anoftalmia causados por déficits de volume orbital ou deslocamento de volume é um método cirúrgico eficaz, confiável e reprodutível.

## INTRODUCTION

Long-term prosthesis users with anophthalmic sockets can develop many problems such as ptosis, upper eyelid sulcus deepening, lower and upper fornix deficiencies, lower eyelid laxity, entropion, ectropion, sphere implant exposure and extrusion, epiphora, and/ or secretion after a period of time^(1^-^4)^. Each of these problems may have particular reasons, but many are due to socket-prosthesis imbalances. Perfect globe-orbit-eyelid integrity, balance, and conformity are important both functionally and cosmetically. The purpose of implants placed in the orbit through traditional evisceration or enucleation surgery and prosthesis placement is to enable such balance^(3)^. However, with time certain changes occur to this balance: orbital fat tissue atrophy and weakening or contraction of extraocular muscles (and other orbital structures) decrease the socket volume. As a result, the socket-prosthesis-eyelid triad compatibility gets deteriorated.

The aim of this study was to retrospectively evaluate the patients who underwent reconstruction with dermofat graft due to anophthalmic socket in terms of anophthalmic socket problems and surgical results.

## METHODS

We retrospectively reviewed the medical records of 21 anophthalmic socket patients treated with dermofat graft implantation surgeries in our clinic between May 2011 and June 2016. We recorded the anophthalmic socket causes, the surgical operations, and the prosthesis duration by each patient.

Ophthalmologists conducted full preoperative ophthalmologic examinations. They documented instances of eyelid problems such as ptosis and upper sulcus deepening related to socket volume deficiency and conjunctival surface insufficiency, upper and lower fornix deficiency, epiphora and burring frequency in a month, socket mobility, lower eyelid laxity, entropion, and ectropion. We recorded orbital and socket anatomies, the presence or absence of orbital spheres (if any), and their localization and material (acrylic or hydroxyapatite) after examining preoperative computerized orbital tomography (CT) images.

The surgeon marked the socket center in the primary gaze position and then determined the socket mobility in the horizontal directions measuring with a ruler. Socket mobility was classified as mild if lower than 2 mm, as moderate from 2 to 5 mm, and as good above 5 mm. After the dermofat graft implantations, the ophthalmologist measured the upper eyelid level in the eye with a prosthesis ruler and assessed increases in 1 mm or more at the upper eyelid level as successful for ptosis compared to the preoperative measurement.

We also evaluated subjective findings such as upper sulcus depth, volume insufficiency, conjunctival surface insufficiency, depth of fornix, and eyelid laxity. The indication for anophthalmic socket reconstruction with dermofat graft in our patients was the presence of problems thought to be due to volume insufficiency.

The patients were followed up one day, one week, one month, three months, six months, and one year after the operations. Following the operations, new prosthesis suitable for the new socket area were implemented in all the patients between the 2^nd^ and 3^rd^ postoperative months. The mean follow-up period was 27.42 ± 16 months (range, 10-62 months). In the final follow-ups, corrected or uncorrected problems were recorded by comparing examination results to those performed preoperatively. All complications that occurred either during the operation or postoperatively were also recorded.

**Figure 1:**
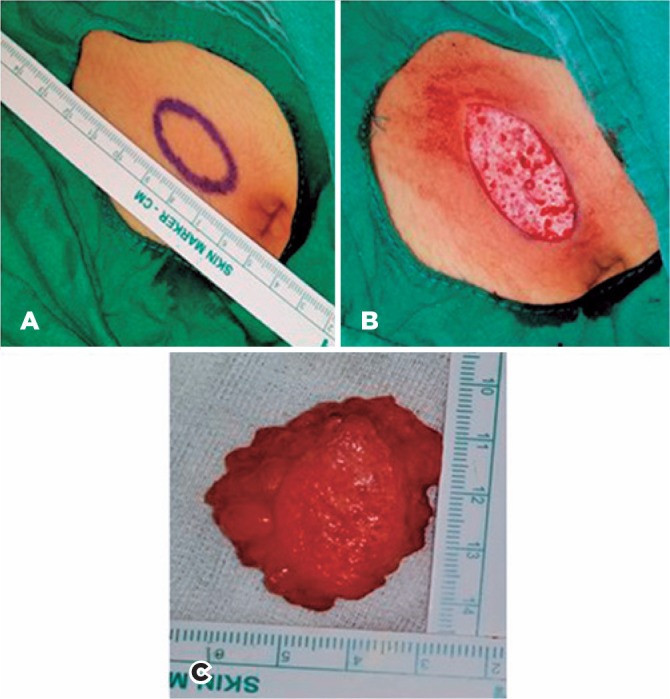
Surgical specimen photographs A) Marked right periumbilical abdomen; B) Epidermis separated from dermis; C) Harvested dermofat graft.

**Figure 2:**
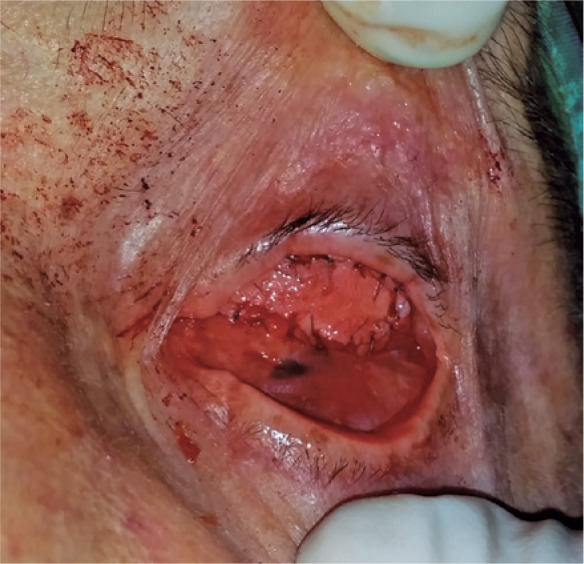
Representative case of a 70-year-old patient with socket volume displacement. The surgeon repaired the volume deficit by placing a dermofat graft in a decentralized (superior) manner.

All patients were operated under general anesthesia. The surgeon chose the right periumbilical area for harvesting each dermofat graft. The operation area was sterilized with antiseptic solution and marked with a sterile pen to determine the dermofat graft amount. Hydrodissections were conducted by injecting isotonic liquid between the epidermis and the dermis. Then, the epidermis was separated from the dermis by incision with a scalpel blade 15. Subsequently, the dermofat graft was reached by deepening the incision. The hair roots in the dermofat graft were electrolyzed. Following bleeding control, the subcutaneous tissue and skin in the abdomen were sutured with 4/0 vicryl and prolene sutures, respectively (Figure 1 A-C). The socket was sterilized with antiseptics and the conjunctiva and Tenon’s capsule were opened with horizontal incisions. The sub-Tenon’s capsule was dissected to reach the scleral tissue surrounding the sphere (in patients with orbital spheres) and the intraorbital fat tissue (in patients without spheres); next a suitable dermofat graft implantation area was formed with dissections. In cases with only volume deficits, the surgeon implanted the dermofat graft centrally, whereas in cases with socket volume displacements, they placed the graft tissue in a decentralized way by shaping the dermofat graft (Figure 2). Tenon’s capsules were sutured to the dermis tissue at 360˚ with 6/0 vicryl sutures. At this stage, to make use of the dermofat graft volume, the conjunctiva was sutured to the dermis tissue at 360˚ in patients with surface deficits, whereas in those cases with no conjunctival surface deficits, the graft tissue was embedded in the socket and the conjunctiva was sutured reciprocally with 6/0 vicryl suture. A conformer was placed and tight bandaging was applied for 3 days.

### Statistical methods

We used the Statistical Package for Social Sciences (SPSS) version 22.0 for Windows (SPSS, Chicago, IL, USA) for statistical analyses. We calculated descriptive statistics for the variables (average, standard deviation, median lowest, highest, frequency, and ratio values). We used the Kolmogorov Smirnov Test to measure the variables distribution and the McNemar test to analyze repetitive measurements.

## RESULTS

We included 16 men and 5 women in this study. Their mean age was 38.3 ± 18.4 years (range, 5-75 years). Thirteen patients had anophthalmic sockets in the right eye and 8 had them in the left eye. Eighteen patients had prosthesis whereas 3 had none. The mean duration of previous prosthesis use was 9.4 ± 6.8 years (range 1-30 years). The surgical histories included 15 patients with evisceration, 5 with enucleation, and 1 with expander implantation.

**Figure 3:**
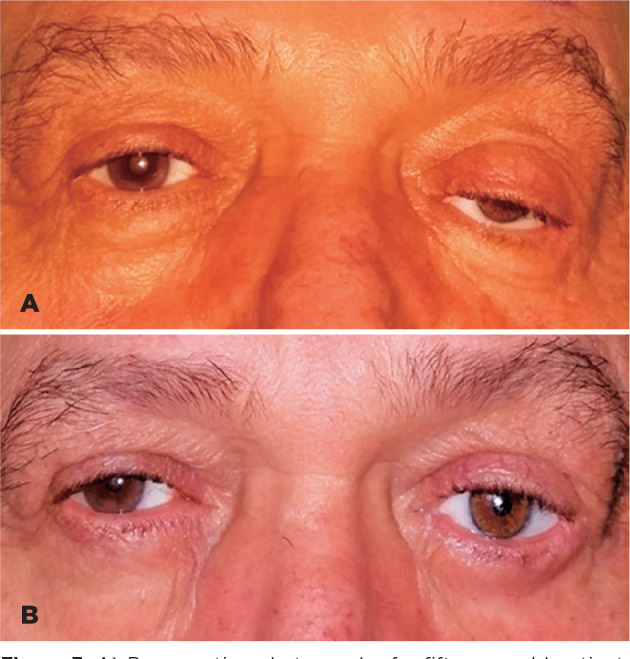
A) Preoperative photograph of a fifty-year old patient, B) Third postoperative month photograph after dermofat graft implantation on the left socket.

After evaluating the preoperative CT images of all patients, we found 13 with orbital spheres and 8 without them. We diagnosed 7 patients as having only volume deficits and 14 as having both volume displacements and deficits. We found inferomedial orbital volume displacements in 9 patients and inferolateral orbital volume displacements in 5. In one patient had an opening in the conjunctiva socket, and three patients had socket extrusions. Preoperatively, 18 patients had ptosis and 15 had also upper sulcus deepenings. We detected lower fornix insufficiencies in 11 patients preoperatively and 6 of them had also lower eyelid laxity. Additionally, two patients had upper fornix deficiencies (Figure 3 A, B).

Preoperatively, 14 patients showed no mobility, whereas 7 showed mild mobility, and all patients had secretion histories requiring topical treatment at least monthly. Surgeons re-operated 9 patients who had incomplete corrections after the dermofat graft implantation at different sessions (6 patients underwent ptosis surgeries, 5 lateral canthal suspensions, 5 mucosal graft lower fornix formations, and 2 upper fornix formations). All patients were able to use prostheses during the postoperative period. One patient had melting of the dermis part of the dermofat graft, but no other complications (Table 1).

**Table 1:** Epidemiological information

	Min-Max	Median	Mean ± S.D n (%)
Age	5 - 75	33	38.3 ± 18.4
Sex			
Male			16 76.2
Female			5 23.8
Duration of prosthesis	1 - 30	10	9.4 ± 6.8
Follow-up period (months)	5 - 62	14	22.8 ± 18.1
Side			
Right			13 61.9
Left			8 38.1
Sphere			
(+)			13 61.9
(-)			8 38.1
Additional surgery			
(+)			9 42.9
(-)			12 57.1
First surgery			
Enucleation			5 23.8
Evisceration			15 71.4
Expander			1 4.8
Preoperative volume			
displacement			
(+)			7 33.3
(-)			14 66.7
Expansion			
(+)			17 81.0
(-)			4 19.0

We detected statistically significant orbital volume increases after the operations (p<0.05). We found significant ptosis recoveries (p<0.05 differences in cases before and after the operations) and similarly significant recoveries in upper sulcus deepening (p<0.05). We found no significant changes in lower eyelid laxity after the operations (p>0.05), but found significant recoveries in lower fornix deficiencies (p<0.05). Also, we found no significant changes in the upper fornix deficiencies after the operations (p>0.05). But, the postoperative secretion rate was lower that the preoperative one (p<0.05). Finally, the prosthesis mobility increased significantly after the operations (p<0.05) (Table 2).

**Table 2:** Statistical assessment of changes after the surgery

		Preoperative		Postoperative		
		n	%	n	%	p-value
Volume	Deficient	20	95.2	0	0.0	0.000
	Full	1	4.8	21	100.0	
Ptosis	(-)	3	14.3	18	85.7	0.000
	(+)	18	85.7	3	14.3	
Lower eyelid laxity	(-)	15	71.4	16	76.2	1.000
	(+)	6	28.6	5	23.8	
Lower fornix deficiency	(-)	10	47.6	16	76.2	0.031
	(+)	11	52.4	5	23.8	
Upper fornix deficiency	(-)	19	90.5	19	90.5	1.000
	(+)	2	9.5	2	9.5	
Secretion rate	(-)	0	0.0	21	100.0	0.000
	(+)	21	100.0	0	0.0	
Motility	(-)	14	66.7	0	0.0	0.000
	(+)	7	33.3	21	100.0	
Upper sulcus deepening	(-)	6	28.6	18	85.7	0.000
	(+)	15	71.4	3	14.3	

Mc Nemar test

## DISCUSSION

The triple balance between socket, prosthesis, and eyelids is important in patients with anophthalmic sockets. In the patients using prosthesis for long terms, orbital volume loss affects both the upper and lower eyelids^(2^-^4)^. Problems such as upper sulcus deepening and ptosis may occur in upper eyelids, and as entropion or ectropion may occur in lower eyelids.

Dermofat grafts are an alternative to solve these orbital socket volume deficit problems. Many techniques have been described for removal and preparation of dermofat grafts^(2^,^5^-^7)^. Dermofat grafts can easily be harvested from the area between the ischial tuberosity and the greater trochanter in the hip or from the lower abdominal area^(5^-^7)^. In our study, we preferred the periumbilical area in abdomen.

All the patients in our study had orbital volume deficiencies before the corrective operations. Some patients also had multiple eyelid problems such as ptosis, upper sulcus deepening, upper and lower fornix deficiency, and lower eyelid laxity. The dermofat graft implantations corrected all the orbital volume deficiencies. Significant postoperative improvements were obtained in terms of ptosis, upper sulcus depth, and lower fornix failure compared to preoperative values. Similarly, Kuzmanović Elabjer et al.^(8)^ reported correcting the deepening of the upper sulcus and volume deficit safely with dermofat grafts in secondary anophthalmic socket reconstructions. Aryasit et al.^(2)^ reported successful use of dermofat grafts in exposure, extrusion, and volume deficits.

An advantage of dermofat grafts is that they can provide a surface increase in patients with socket surface and fornix deficiencies^(9)^. In patients with lower and upper fornix deficiencies, the socket conjunctiva can be shifted to the fornixes using the volume of the dermofat graft, which can be fixed with deepening sutures. The problem of lower and upper fornix deficiencies can be solved without a second mucosal graft. In our study, although we achieved some success in patients with lower fornix deficiency, we did not succeed in correcting upper fornix deficiencies. Therefore, we formed a fornix with oral mucosal graft in a secondary operation in patients with upper and/or lower fornix deficiencies.

Prosthesis movement is a leading wish of patients with anophthalmic sockets. However, prosthesis mobi lity is still less than satisfactory^(4^,^10^-^13)^. Changes in the socket along with volume losses affect the existing socket movement. In our study we measured and compared preoperative and postoperative socket movements and obtained a significant increase in postoperative socket movement. We attribute this to the correction in the socket and prosthesis disbalance due to the dermofat graft.

Many of our patients had either orbital volume deficiencies or volume displacements (toward inferior, inferomedial, or inferolateral locations) along with the volume deficiency. This causes incompatibility between the prosthesis and the socket interface, and leads to secretion or infection. To us, one advantage of the dermofat graft is that more volumes can be provided to the areas with higher regional volume loss by shaping the graft tissue and implanting it in a decentralized position. In our study, we found significant decreases in problems (such as secretion, infection and epiphora) that had been frequent due to socket-prosthesis surface irregularity before the operation.

Dermofat grafts in oculoplastic operations have been carried out to complete the anophthalmic socket volumes in primary or secondary procedures^(9^,^14^-^17)^. But, the widespread use of synthetic spheres in the oculoplastic practice has limited the primary use of dermofat grafts. In some studies, the primary use of dermofat grafts is better than the secondary use, in terms of tissue resorption, and it even causes excessive growth in children^(18^,^19)^. We observed tissue resorptions in dermofat grafts during the follow-up period but not enough to affect the surgical success. In our study, only one patient was diagnosed as having dermis melting of the dermofat graft in the postoperative term. During the follow-ups of this patient, no secondary operations were needed as the conjunctival tissue proceeded on the graft and covered it. We found no cases of hairing, infection, or socket exposures.

One of the limitations of our study was the small number of patients participating. Another limitation had to do with the unavailable quantitative measurements of volume and fornix insufficiencies, and eyelid laxity.

As a conclusion, many problems due to orbital volume insufficiency may develop in patients with anophthalmic sockets using prostheses for a long time. Volume insufficiency should be corrected before intervening eyelid problems in anophthalmic socket patients. Dermofat graft implants can correct both volume losses and some eyelid problems. Thus, dermofat graft implantation is an effective, reliable, surgical alternative.
